# Associations Between Mediterranean Diet, Processed Food Consumption, and Symptoms of Anxiety and Depression: Cross-Sectional Study Among Israeli Adults

**DOI:** 10.3390/foods14091485

**Published:** 2025-04-24

**Authors:** Nourit Houminer-Klepar, Keren Dopelt

**Affiliations:** Department of Public Health, Ashkelon Academic College, Ashkelon 78211, Israel; nhouminer@gmail.com

**Keywords:** Mediterranean diet, processed food, anxiety, depression, sleep quality, cross-sectional study, Israel

## Abstract

Introduction: Mental health disorders, particularly anxiety and depression, contribute significantly to the global disease burden. Growing evidence suggests that dietary patterns play a crucial role in mental health outcomes. Objectives: This cross-sectional study examines the associations between adherence to a Mediterranean diet, processed food consumption, and symptoms of anxiety and depression among Israeli adults. Additionally, it investigates whether sleep quality mediates these relationships. Methods: A total of 303 participants completed an online survey assessing demographic variables, health-related behaviors, dietary patterns, sleep quality and duration, and mental health indicators. Results: Pearson correlations revealed significant associations between dietary patterns and mental health symptoms. Higher Mediterranean diet adherence was associated with lower anxiety and depression scores, whereas greater processed food consumption was linked to increased psychological distress. Hierarchical regression analyses showed that dietary patterns significantly predicted anxiety and depression symptoms, even after controlling for demographic and health-related factors. However, sleep quality did not mediate these relationships, suggesting independent effects of diet and sleep on mental health. Conclusions: These findings highlight the potential of dietary modifications as a complementary approach to mental health management, though generalizability is limited by our predominantly female, Jewish Israeli sample studied during a period of conflict. Integrating nutritional assessments into mental health care could enhance treatment strategies. Future research should explore longitudinal and interventional approaches to establish causal relationships between diet quality and mental health outcomes. This study highlights the clinical and public health relevance of dietary assessment in mental health evaluations and supports the development of integrated, nutrition-informed interventions to enhance psychological well-being.

## 1. Introduction

Depression and anxiety affect millions globally, with major depressive disorder (MDD) having a global prevalence of 332 million cases in 2021, with an age-standardized prevalence rate of 4006.8 per 100,000 population [1], and anxiety disorders affecting approximately 359 million people worldwide, with an age-standardized prevalence rate of 4421.9 per 100,000 population [2]. These disorders are more prevalent in women and rank among the top 25 causes of global disease burden, highlighting their significance as major public health challenges [3]. The COVID-19 pandemic further exacerbated this issue, leading to a substantial rise in mental health disorders. Major depressive disorders increased by 27.6%, and anxiety disorders by 25.6%, contributing an additional 53.2 million and 76.2 million cases globally, respectively [4]. This global increase in mood disorders emphasizes the importance of exploring modifiable lifestyle factors, such as diet, in mental health prevention and treatment.

Depression is characterized by persistent sadness, anhedonia, and impaired functioning, while anxiety manifests as excessive worry and physiological arousal, including tachycardia and respiratory changes [5]. These conditions have multifactorial etiologies involving genetic, neurobiological, environmental, and psychological components [6] and can be addressed through psychotherapeutic approaches, pharmacological interventions, and lifestyle modifications, including dietary changes [7].

Dietary modulation is gaining recognition as an effective adjunct to mental health treatments. Its advantages lie in being a low-cost, low-risk, and widely accessible intervention that may appeal to individuals who prefer non-invasive or preventive approaches [8], with growing evidence supporting the complex relationship between nutritional patterns and depression and anxiety [7]. Research consistently demonstrates that Mediterranean-style dietary patterns, characterized by abundant plant foods, lean proteins, healthy fats, and minimal ultra-processed foods, are associated with reduced depression risk and improved symptoms in clinical and non-clinical populations [9]. Conversely, Western dietary patterns high in processed foods, refined carbohydrates, and foods with high dietary inflammatory potential correlate with increased depression and anxiety symptoms [10,11].

The Mediterranean diet is widely recognized for its mental health benefits and its therapeutic role in managing chronic diseases such as obesity, diabetes, and cardiovascular conditions. Rich in anti-inflammatory and antioxidant components, this dietary pattern has been associated with improved cardiometabolic markers, reduced systemic inflammation, and better glycemic control. During the COVID-19 pandemic, when food-related stress and unhealthy eating behaviors were particularly prevalent, adherence to the Mediterranean diet emerged as a protective factor for physical and psychological well-being. Recent studies have highlighted its relevance in reducing COVID-19-related health risks, supporting immune function, and improving outcomes in patients with comorbid conditions [12,13,14]. These findings highlight the Mediterranean diet’s role in enhancing mental health and supporting effective treatment outcomes, both generally and specifically during global crises.

Multiple biological mechanisms explain the link between diet and mental health, including systemic inflammation, oxidative stress, and bidirectional communication via the gut–brain axis, where dietary components influence gut microbiota composition and function, affecting neurotransmitter production and neural signaling [15,16]. A Western diet rich in processed foods increases chronic inflammation in the body and leads to the release of inflammatory substances (cytokines) that cross the blood–brain barrier and contribute to depression and anxiety symptoms [17]. Conversely, diets rich in anti-inflammatory compounds, antioxidants, and essential nutrients can promote optimal neurotransmitter function, reduce systemic inflammation, and support beneficial gut microbiota, potentially improving mood regulation and the stress response [18,19]. Several biomarker studies support this mechanism, demonstrating that higher Mediterranean diet adherence is associated with lower levels of inflammatory markers such as C-reactive protein (CRP), interleukin-6 (IL-6), and tumor necrosis factor-alpha (TNF-α) [18]. Western diets are also typically deficient in protective nutrients, including antioxidants, vitamins (such as B complex vitamins), and various minerals (such as zinc, iron, and magnesium) [20,21,22,23], dietary fiber, and omega-3 fatty acids, which collectively help regulate inflammation, maintain intestinal barrier integrity, support a healthy gut microbiome, and combat oxidative stress in both the gut and neural tissues [16,24]. These nutritional deficiencies compromise the optimal functioning of neuronal pathways, affecting neurotransmitter synthesis, including serotonin, which is heavily implicated in mood regulation and depression, and impairing immune regulation, all of which are critical for mental health.

Importantly, heightened inflammation is not only linked to mood disorders but also appears to influence treatment outcomes. Patients with depression and anxiety often exhibit elevated levels of inflammatory cytokines, and those with higher inflammation show reduced responsiveness to conventional psychopharmacological treatments, suggesting that inflammation may be a key factor in treatment resistance [25].

Emerging evidence from interventional studies strengthens the link between diet and mental health by demonstrating a causal relationship between dietary modifications and reductions in depressive symptoms. Randomized controlled trials indicate that adherence to Mediterranean diet interventions leads to clinically meaningful improvements in depression scores, with moderate to large effect sizes, even among individuals diagnosed with depression. A notable example of such a study is the SMILES trial, where the control group received structured social support, an intervention known for its mental health benefits [26]. Importantly, these benefits are most pronounced when dietary changes are implemented with structured, professional support, highlighting the potential of nutritional psychiatry as an adjunct to conventional treatments [7]. Beyond individual trials, large-scale meta-analyses provide compelling support for the role of diet in mental health. For instance, high consumption of ultra-processed foods has been associated with a significantly increased risk of developing mental disorders (53%), depression (44%), and anxiety (48%) [27]. Conversely, transitioning to a Mediterranean diet has been linked to significant reductions in depressive symptoms, reinforcing its potential as a preventive and therapeutic strategy [28]. These findings highlight the importance of integrating dietary interventions into mental health care, particularly given the substantial comorbidity between mood disorders and metabolic health issues.

Given the accumulating evidence linking dietary patterns with mental health outcomes, further research is needed to understand these associations across diverse populations and cultural contexts. The primary objective of this study was to investigate the relationship between adherence to a Mediterranean diet and symptoms of anxiety and depression among Israeli adults. The secondary objective was to examine the association between processed food consumption and symptoms of anxiety and depression and to explore whether sleep quality moderates these relationships. While this study was conducted within the Israeli population—one experiencing heightened mental health challenges, as evidenced by a 20% increase in anti-anxiety medication use following the Iron Swords War in October 2023 [29]—its findings have broader relevance for understanding how dietary patterns influence mental health. By examining these associations, this research contributes to the growing global body of evidence on modifiable lifestyle factors that shape mental health across diverse cultural and societal contexts.

## 2. Methods

### 2.1. Procedure

The current study was a cross-sectional, descriptive study employing survey methodology to examine the relationships between dietary patterns and health-related behaviors, including sleep quality and duration, physical activity, and mental health outcomes. Approval for the study was obtained from the Ashkelon Academic College Ethics Committee (approval #54-2024). The survey questionnaire was conducted via Qualtrics (Qualtrics, Provo, UT, USA) and distributed electronically through multiple digital channels, including messaging platforms (WhatsApp, Menlo Park, CA, USA), social media networks (Instagram, Menlo Park, CA, USA and Facebook, Menlo Park, CA, USA), and email distribution lists. This multi-platform recruitment strategy was employed to maximize reach and diversity within our target population. Participants accessed the survey via a secure link that directed them to the questionnaire platform. The study took place between 29 December 2024 and 1 March 2025. The survey introduction page outlined the study’s objectives and assured participant anonymity. Completion of the questionnaire served as documentation of participants’ voluntary informed consent. Participants retained the right to withdraw at any point during the survey process, and question responses were not mandatory.

A total of 303 responses were recorded, of which n = 253 completed the full questionnaire. Participants with missing data (n = 50) were compared to those with complete data across all demographic and outcome variables using independent samples t-tests for continuous variables and chi-square tests for categorical variables. No significant differences were found between groups, suggesting data were missing at random.

The study was reported in accordance with the STROBE guidelines for cross-sectional research [30] (see Appendix A).

### 2.2. Tools

Questionnaires were submitted electronically and consisted of four parts: demographic and health-related data, including age, gender, relationship status (in a relationship—Yes/No), socioeconomic status (above average, average, or below average), education, and religion, and health-related information, including weight and height, to calculate body mass index, or BMI (Kg/m^2^), physical activity frequency, and sleep duration and quality. Diet-related questions included adherence to the Mediterranean diet and processed food consumption; and mental-health-related questions included depressive symptoms and anxiety symptoms.

Physical activity was assessed using a single-item measure evaluating participants’ physical activity levels during the previous week. This measure demonstrates high reliability with a correlation of 0.86 and concurrent validity (compared to the Global Physical Activity Questionnaire [GPAQ]) with a correlation of r = 0.53 [31]. Participants were asked: “During the past 7 days, on how many days did you perform physical activity for at least 30 min per day?”

Sleep quality and duration were evaluated using the Pittsburgh Sleep Quality Index (PSQI), which assesses sleep patterns over the preceding month. This study specifically utilized the sleep quality and duration components, following the methodology of Buysse et al. [32], who reported an internal consistency (Cronbach’s alpha) of 0.83 [32]. The combined score for quality and duration of sleep ranged from 0 to 6, with 0 representing optimal sleep (very good subjective quality and >7 h duration) and 6 indicating the poorest sleep (very bad subjective quality and <5 h duration). A previously translated and validated Hebrew version of the PSQI demonstrated an internal consistency of Cronbach’s alpha of 0.72 [33].

Diet quality was assessed using the Israeli Mediterranean diet screener (IMEDAS) [34], a tool designed to evaluate adherence to the Mediterranean dietary pattern. The index comprises 17 questions measuring Mediterranean diet adherence through consumption frequency of characteristic foods (olive oil, vegetables, fruits, whole grains, legumes, and fish) and non-characteristic foods (red meat, sweet pastries, and salty snacks). Each question awards one point for meeting specific criteria, either consuming sufficient amounts of characteristic Mediterranean foods or limiting consumption of non-characteristic foods like processed items, and zero points for non-adherence. Scores range from 0–17, with higher values indicating greater Mediterranean diet adherence. A score of 9 or above represents good adherence. For this study, the alcohol consumption question was omitted.

The processed food scale was developed using four components from the IMEDAS questionnaire (sweet pastries, salty pastries, salty snacks, and processed meats). Rather than employing the traditional binary coding system (0 or 1), a quantitative approach based on serving sizes consumed was implemented. The serving sizes across all four components were summed to calculate total processed food consumption, providing a continuous measure where higher totals indicated greater consumption. This approach allowed for a more nuanced assessment of processed food intake, accounting for the quantity consumed. Sweetened beverages were excluded from this scale due to extreme outliers in individual consumption patterns (with some participants reporting more than 70 weekly servings per person) that significantly skewed the distribution.

Anxiety symptoms were assessed using the GAD-7 questionnaire: The GAD-7 is a validated screening and assessment tool for measuring the severity of anxiety symptoms. The questionnaire was developed by Spitzer et al. [35] and had been translated and validated in Hebrew [35]. The questionnaire consisted of 7 items describing common symptoms of generalized anxiety disorder, where respondents were asked to rate the frequency of symptom occurrence in the past two weeks on a 4-point Likert scale (0 = “not at all” to 3 = “nearly every day”). The score range is from 0 to 21, with higher scores indicating higher levels of anxiety [35]. The internal reliability of the questionnaire was found to be high, with Cronbach’s alpha of 0.90.

Depressive symptoms were measured using the Patient Health Questionnaire-9 (PHQ-9): The PHQ-9 is a validated screening and assessment tool for measuring the severity of depressive symptoms. The questionnaire was developed by Kroenke et al. [36] and had been translated and validated in Hebrew [36]. The questionnaire consisted of 9 items based on the criteria for diagnosing major depression according to the DSM, where respondents were asked to rate the frequency of symptom occurrence in the past two weeks on a 4-point Likert scale (0 = “not at all” to 3 = “nearly every day”). The score range is from 0 to 27, with higher scores indicating higher levels of depression [36]. The internal reliability of the questionnaire was found to be high, with Cronbach’s alpha of 0.89

### 2.3. Statistical Analysis

All analyses were conducted using SPSS version 27 (IBM, Armonk, NY, USA), with statistical significance set at *p* < 0.05. Missing data were handled using listwise deletion after confirming no significant differences between participants with complete versus incomplete data across all study variables. First, descriptive statistics of participant characteristics were conducted, including socio-demographic information, lifestyle and health-related information (BMI, physical activity frequency, and sleep quality and duration), mental health indicators (anxiety and depression symptoms), and dietary pattern indicators (Mediterranean diet adherence and consumption of processed foods), and were assessed for all participants (n = 303). Second, Pearson correlations were performed to explore relationships between dietary patterns, mental health symptoms, and health-related behaviors. Third, we conducted independent samples t-tests to examine potential differences in eating patterns between high and low anxiety/depression, as determined by the cut points of GAD-7 ≥ 8 for anxiety [37] and PHQ-9 ≥ 10 for depression [38]. Fourth, to examine the relationships between dietary patterns, health-related behaviors, and depression and anxiety symptoms, separate hierarchical regression analyses were conducted for depression (PHQ-9) and anxiety (GAD-7) symptoms as outcome variables. Variables were entered into four sequential blocks: (1) demographic factors (age, gender, socioeconomic status, and relationship status); (2) health-related behaviors (BMI and physical activity); (3) dietary patterns (Mediterranean diet adherence and processed food consumption); and (4) sleep quality. Sleep quality was entered in the final block due to its known strong association with mental health outcomes and to examine its independent contribution beyond dietary patterns. For each block, R^2^, changes in R^2^, 95% confidence intervals (CI), significance, and standardized coefficients were analyzed to determine each predictor’s significance and relative contribution. Potential confounding variables were included as covariates based on established relationships with both dietary patterns and depression in prior research. We included age and gender as demographic factors that influence dietary choices and depression risk [39]. Socioeconomic status was included due to its well-documented impact on both food access and mental health outcomes [40]. BMI was included as it established bidirectional relationships with both dietary intake and depression [41,42]. Physical activity was incorporated as a covariate due to its independent effects on both nutritional choices and mood regulation through neurobiological pathways [43,44,45]. Relationship status was included based on prior evidence suggesting that being in a relationship affects both eating behaviors through shared meal patterns and food environments [46] and provides social support that influences depression risk [47,48]. Fifth, mediation analyses were conducted using the PROCESS macro for SPSS [49] to examine whether sleep quality and duration mediated the relationship between Mediterranean diet adherence and mental health outcomes (depression and anxiety symptoms). Simple mediation models were specified with 5000 bootstrap samples to estimate direct and indirect effects with 95% confidence intervals. Mediterranean diet scores were entered as the independent variable (X), mental health measures (depression/anxiety) as the dependent variables (Y), and sleep parameters as the potential mediators (M).

## 3. Results

### 3.1. Sample Characteristics

Sample demographics, participants’ health-related factors, mental health, and dietary pattern indicators are shown in Table 1. The study sample (N = 303) consisted predominantly of Jewish (98.3%) females (76.9%) with an age range of 20–74 and a mean age of 32.6 years (SD = 11.1). Most participants reported average or above-average socioeconomic status (86.7%), and over half were in a relationship (56.8%). Health indicators revealed that participants had a mean BMI of 25.5 (SD = 6.2), with the majority (61.7%) engaging in physical activity at least once weekly, though over a third (38.3%) reported no regular physical activity. Mental health measures indicated moderate levels of depression (DHQ-9 M = 11.5, SD = 6.8) and anxiety (GAD-7 M = 10.1, SD = 5.5). The dietary assessment showed moderate-to-low adherence to the Mediterranean diet (IMEDAS M = 6.2, SD = 2.2) alongside substantial processed food consumption (M = 7.7, SD = 5.1).

### 3.2. Relationships Between the Study Variables

Pearson correlations were conducted to explore relationships among dietary patterns (adherence to the Mediterranean diet and consumption of processed food), mental health symptoms (anxiety and depression), health-related behaviors (physical activity frequency and sleep quality and duration), and age. As shown in Table 2, Mediterranean diet adherence showed significant negative correlations with both anxiety (r = −0.202, *p* = 0.001) and depression (r = −0.176, *p* = 0.005) and was negatively associated with processed food consumption (r = −0.438, *p* < 0.001). Conversely, processed food consumption demonstrated significant positive correlations with anxiety (r = 0.141, *p* = 0.026) and depression (r = 0.150, *p* = 0.018). Sleep disturbance was significantly associated with both anxiety (r = 0.148, *p* = 0.014) and depression (r = 0.227, *p* < 0.001), with a stronger relationship observed for depression. Physical activity frequency was positively correlated with Mediterranean diet adherence (r = 0.272, *p* < 0.001) and negatively associated with sleep disturbance (r = −0.136, *p* = 0.022). Age demonstrated significant negative correlations with anxiety (r = −0.219, *p* < 0.001) and depression (r = −0.237, *p* < 0.001), suggesting that younger individuals reported higher levels of anxiety and depression symptoms.

Independent samples t-tests were conducted to compare demographic factors, health behaviors, and sleep quality and duration between participants with high versus low anxiety (using the established GAD-7 cut-off score of ≥8) [36]. Results shown in Table 3 revealed that participants with high anxiety were significantly younger and reported lower Mediterranean diet adherence compared to the low-anxiety group (*p* = 0.002 and *p* = 0.008, respectively). Additionally, the high-anxiety group showed trends toward higher processed food consumption and poorer sleep quality and duration, though these differences were marginally non-significant (*p* = 0.069 and *p* = 0.080, respectively). No significant differences were observed for physical activity or BMI between groups.

Similarly, when comparing participants with high versus low depression (using the established PHQ-9 cut-off score of ≥10) [38], those with higher depression symptoms were significantly younger (*p* = 0.003) and reported lower Mediterranean diet adherence (*p* = 0.025) than participants with low depression symptoms. Unlike the anxiety comparison, the difference in sleep quality and duration between depression groups reached statistical significance (*p* < 0.001), with the high-depression group reporting substantially poorer sleep. While the high-depression group also consumed more processed foods than the low-depression group, this difference did not reach statistical significance (*p* = 0.151). Similar to the anxiety analysis results, no significant differences in physical activity or BMI were observed between the low and high depression groups.

### 3.3. Regression Models

In the hierarchical regression analyses (Table 4 for anxiety, Table 5 for depression), dietary patterns (Model 3) collectively contributed significant additional variance in both anxiety symptoms (ΔR^2^ = 0.025, *p* = 0.035) and depression symptoms (ΔR^2^ = 0.022, *p* = 0.045) beyond demographics and health-related behaviors. However, when examined simultaneously within the models, neither Mediterranean diet adherence nor processed food consumption reached clear statistical significance individually, despite their collective significance. For depression, processed food consumption approached significance (β = 0.130, *p* = 0.053). The addition of sleep duration and quality (Model 4) significantly improved model fit for both anxiety (ΔR^2^ = 0.014, *p* = 0.046) and depression (ΔR^2^ = 0.045, *p* < 0.001), with a notably stronger effect observed for depression symptoms.

When examining dietary patterns separately in relation to anxiety symptoms, individual regression models revealed that both dietary patterns maintained significance even after controlling sleep quality. The Mediterranean diet model showed that higher adherence was significantly associated with reduced anxiety symptoms (β = −0.146, *p* = 0.022) after controlling demographic factors, health-related behaviors, and sleep quality. Similarly, processed food consumption was significantly associated with increased anxiety symptoms (β = 0.142, *p* = 0.023) independent of sleep quality. Mediterranean diet adherence explained 2.1% of the unique variance (ΔR^2^ = 0.021, *p* = 0.016) in anxiety symptoms, while processed food consumption accounted for 1.9% (ΔR^2^ = 0.019, *p* = 0.024). In both models, sleep quality contributed approximately 1.5% additional explained variance, demonstrating that dietary patterns and sleep quality represent distinct pathways associated with anxiety symptoms.

Similar findings emerged when examining dietary patterns separately in relation to depression symptoms. In the Mediterranean diet model, higher adherence was significantly associated with reduced depression symptoms (β = −0.126, *p* = 0.043), explaining 1.4% unique variance (ΔR^2^ = 0.014, *p* = 0.043) after controlling for demographics and health-related behaviors. After adding sleep quality in the final block, Mediterranean diet adherence approached but no longer reached statistical significance (β = −0.112, *p* = 0.065). Likewise, processed food consumption was significantly associated with increased depression symptoms (β = 0.146, *p* = 0.017), accounting for 2.0% of the unique variance (ΔR^2^ = 0.020, *p* = 0.017), and remained significant after controlling for sleep quality (β = 0.146, *p* = 0.014). Sleep quality demonstrated a stronger relationship with depression than with anxiety, contributing 4.5–4.6% additional explained variance (*p* < 0.001) in both dietary models. Formal mediation analyses, however, did not support sleep quality and duration as a significant mediator of the relationship between the Mediterranean diet and either depression (indirect effect = −0.088, 95% CI [−0.218, 0.012]) or anxiety (indirect effect = −0.045, 95% CI [−0.121, 0.006]). These findings suggest that dietary patterns and sleep quality represent distinct pathways to mental health outcomes rather than a causal sequence where diet influences mental health through sleep-related mechanisms.

## 4. Discussion

The present study examined the relationships between dietary patterns, health-related behaviors, and mental health outcomes among 303 adult participants in Israel. Our results demonstrated significant associations between dietary patterns and mental health symptoms. First, our results revealed that both Mediterranean diet adherence and processed food consumption demonstrated relationships with anxiety and depression symptoms when examined in separate models. Mediterranean diet adherence was inversely associated with both anxiety and depression, consistent with previous research suggesting the protective effects of this dietary pattern on mental health [50,51]. Conversely, processed food consumption showed positive associations with anxiety and depression symptoms, aligning with a growing body of evidence linking ultra-processed food intake to poorer mental health outcomes [7,10,26,27]. Highly processed foods, characterized by a high glycemic load, unhealthy fats, artificial additives, and low fiber content, are known to contribute to systemic inflammation, oxidative stress, and gut microbiota dysbiosis, all of which have been implicated in the pathophysiology of mood disorders [17]. Notably, the pro-inflammatory nature of processed foods may contribute to treatment-resistant depression, as individuals with higher baseline inflammation levels tend to exhibit poorer responses to conventional antidepressant therapies [25,52,53]. Interestingly, when both dietary patterns were included simultaneously in our models, their individual effects were attenuated, suggesting shared variance between these dietary measures. This finding emphasizes the challenge of isolating the effects of specific dietary patterns, as food consumption behaviors often cluster together. For example, individuals who adhere more to Mediterranean diet components typically consume fewer processed foods [54,55].

Our findings regarding the negative association between Mediterranean diet adherence and mental health symptoms align with established mechanisms involving antioxidants, anti-inflammatory compounds, and essential micronutrients that support neurotransmitter synthesis and reduce systemic inflammation [18,19,56,57].

The inclusion of the sleep quality and duration scale in our analyses revealed several important insights. Sleep quality demonstrated significant associations with both anxiety and depression symptoms, with a notably stronger relationship observed for depression. This aligns with the extensive literature documenting the bidirectional relationships between sleep disturbances and depressive symptoms [58,59]. Compared to previous studies, the present research offers several advantages, including the simultaneous examination of both adherence to the Mediterranean diet and consumption of ultra-processed foods within the same model, the inclusion of multiple behavioral and demographic covariates, and the focus on an understudied, community-based Israeli adult population. While sample characteristics limit generalizability, these elements contribute to our understanding of dietary patterns and mental health outcomes in specific cultural contexts

We did not find evidence that sleep quality mediates the relationship between dietary patterns and mental health outcomes. Rather, our findings suggest that dietary patterns and sleep quality represent distinct pathways to mental health. The attenuation of the Mediterranean diet effect on depression after controlling for sleep quality, coupled with the persistence of the processed food effect, possibly suggests differential mechanisms through which these dietary factors might influence mental health. However, given that sleep duration and quality were self-reported, future studies using objective sleep measures (e.g., actigraphy and polysomnography) may offer more definitive conclusions on this potential interaction.

Interestingly, while Mediterranean diet adherence was associated with improved mental health outcomes [60,61], its effect on depression symptoms was attenuated after controlling for sleep quality [62]. This suggests that sleep may partially explain the protective effects of healthy eating on mood but does not fully account for the observed relationships [62]. By contrast, processed food consumption remained a robust predictor of depressive symptoms, even after adjusting for sleep quality, indicating that the detrimental effects of poor diet on mental health may operate through pathways distinct from those of sleep disturbances [63].

Several demographic and health-related factors emerged as significant predictors of mental health outcomes. Younger age was associated with higher levels of anxiety and depression symptoms, a finding consistent with epidemiological data indicating that mental health disorders disproportionately affect younger adults [64,65]. This may reflect greater socioeconomic stressors, lifestyle instability, and increased exposure to digital stressors (e.g., social media use) in younger populations [66,67,68,69,70].

Relationship status also played a role, with individuals in a relationship reporting lower levels of anxiety and depression. This aligns with research highlighting the protective effects of social support and intimate relationships on psychological well-being [47,48]. However, the extent to which relationship status interacts with dietary habits remains unclear and warrants further investigation.

Interestingly, physical activity frequency was not a significant predictor of mental health symptoms in our models, despite substantial evidence supporting its benefits for psychological well-being [45]. One possible explanation is that the measure used in this study, a single-item self-report assessment, may not have adequately captured exercise intensity, duration, or adherence to structured physical activity routines.

These findings have important implications for both clinical practice and public health policy. Given the growing recognition of lifestyle medicine as an adjunct to conventional psychiatric treatment, healthcare providers should consider incorporating dietary assessments and interventions into mental health care. Encouraging adherence to the Mediterranean diet and reducing processed food consumption could serve as a low-risk, cost-effective strategy to improve psychological well-being, particularly for individuals with mild-to-moderate mood disorders.

At the policy level, these results support nutritional education initiatives aimed at promoting whole-food-based diets and limiting ultra-processed food availability, particularly in schools, workplaces, and healthcare settings. Given the observed mental health benefits associated with a Mediterranean-style diet, public health campaigns should emphasize the psychological advantages of balanced eating patterns alongside traditional physical health benefits (e.g., cardiovascular protection and metabolic regulation).

Recent evidence suggests that combining dietary intervention with other treatment modalities may yield additive or synergistic effects. A randomized controlled trial conducted among postmenopausal women with obesity demonstrated that a combined dietary and physical–cognitive exercise intervention significantly improved executive function, adiponectin levels, and lipid profiles [71]. Similarly, research involving obese adolescents showed that integrating exercise and dietary changes significantly enhanced cognitive and physical self-control and reduced BMI [72]. These findings support the notion that dietary modification may serve as an effective adjunct therapy to psychological and physical interventions, especially within lifestyle-focused treatment frameworks.

### 4.1. Study Limitations

This study has several limitations that should be acknowledged. First, its cross-sectional design prevents us from drawing causal inferences about the relationships between dietary patterns, sleep quality, and mental health outcomes. While our findings suggest associations between Mediterranean diet adherence, processed food consumption, and symptoms of anxiety and depression, we cannot determine whether dietary habits influence mental health or if individuals experiencing psychological distress are more likely to adopt poorer eating behaviors. Future longitudinal studies and randomized controlled trials are needed to establish directionality and causality. Second, self-report measures were used to assess dietary intake, sleep quality, and mental health symptoms. While validated instruments were employed, self-reported dietary data may be subject to recall bias and social desirability bias, potentially leading to underreporting or overreporting of specific food groups. Another limitation is the lack of a comprehensive dietary assessment. While we examined Mediterranean diet adherence and processed food consumption, other nutritional factors, such as specific micronutrients (e.g., omega-3 fatty acids, vitamin D, and B vitamins) and macronutrient composition, were not directly analyzed. These components may play crucial roles in diet–mental health relationships. A critical limitation concerns the generalizability of our findings. Our sample characteristics substantially limit the external validity of our results. The study population was predominantly female (76.9%) and Jewish (98.3%), with a relatively young mean age of 32.6 years. This demographic composition raises important questions about the applicability of our findings to males, non-Jewish populations, and older adults. The biological and psychological mechanisms linking diet to mental health may function differently across gender, cultural background, and age due to hormonal variations, different stress responses, and age-related metabolic changes. Additionally, selection bias may have drawn participants already interested in nutrition–mental health connections. While our findings provide valuable insights into dietary and mental health patterns, caution should be exercised when extrapolating these results to populations with different demographic and cultural characteristics.

### 4.2. Perspective for Clinical Practice

The findings of this study provide practical implications for clinical and preventive mental health care. Given the observed associations between dietary patterns and symptoms of anxiety and depression, mental health practitioners should consider incorporating basic nutritional screening into routine assessments. Identifying individuals with low adherence to healthy dietary patterns, particularly those consuming high levels of processed foods, may support early intervention efforts.

While nutritional counseling is not traditionally part of psychiatric care, these results emphasize the potential of collaborative, interdisciplinary models involving dietitians, psychologists, and primary care providers. Such integration may be especially beneficial for patients with mild-to-moderate symptoms who are seeking non-pharmacological or adjunctive options. Furthermore, promoting adherence to Mediterranean-style diets may be a low-risk, cost-effective tool to enhance psychological well-being and reduce reliance on medication.

In clinical practice, interventions aimed at modifying diet should be tailored to the individual’s preferences, cultural background, and readiness for change. Educational strategies, motivational interviewing, and structured dietary programs may help translate evidence into action. Additionally, raising healthcare professionals’ awareness of food–mood connections could promote holistic treatment plans addressing both mental and physical health.

## 5. Conclusions

This study contributes meaningful evidence to the growing body of research examining the relationships between dietary patterns and mental health outcomes. Our findings revealed significant associations between Mediterranean diet adherence, processed food consumption, and symptoms of anxiety and depression among Israeli adults, which persisted after controlling for relevant demographic and health-related confounders. The observed inverse relationship between Mediterranean diet adherence and mental health symptoms, coupled with the positive association between processed food consumption and psychological distress, aligns with the emerging literature in nutritional psychiatry suggesting diet quality as a modifiable risk factor for mental health disorders.

Our analyses indicate that dietary patterns represent important pathways to mental health outcomes, with sleep duration and quality appearing as a distinct contributing factor rather than a mediating mechanism. This emphasizes the potential value of intervention approaches primarily targeting nutritional habits, while acknowledging the independent contribution of sleep to mental well-being.

These findings may support the integration of nutritional assessment and guidance into mental health treatment protocols. Healthcare providers should consider evaluating dietary patterns as part of comprehensive mental health assessments and incorporate evidence-based nutritional recommendations into treatment plans, particularly for individuals presenting with symptoms of anxiety and depression. In addition, it may assist health policymakers in promoting policies that support nutritional guidance, such as promoting Mediterranean dietary patterns and discouraging excessive consumption of processed foods, to prevent and support mental health issues, specifically anxiety and depression. While this study was conducted in Israel during a period of conflict, our findings contribute to our understanding of diet–mental health relationships, though generalizability to populations with different demographic and cultural characteristics should be approached with caution. In a time when mental health disorders are on the rise globally, dietary interventions represent a promising, low-risk, and accessible strategy for promoting psychological well-being. This study contributes to the growing evidence supporting the relationship between dietary patterns and mental health outcomes, advocating for a holistic, lifestyle-based approach to mental health care. Future research should focus on longitudinal designs to track changes over time and establish causal relationships between dietary factors and mental health outcomes. Additional interventional studies are needed to test the effectiveness of specific dietary interventions or the elimination of processed foods in improving mental health.

## Figures and Tables

**Table 1:** Demographics, health-related factors, mental health, and dietary pattern indicators, with % and number (n = 303).

Variables	Mean/N	SD or %N
**Demographics**		
Age (n = 303)	32.6	11.1
Gender (n = 303)		
Female	n = 233	76.9%
Male	n = 70	231%
Socioeconomic status (n = 301)		
Below average	n = 40	13.3%
Average	n = 174	57.8%
Above average	n = 87	28.9%
Relationship status (n = 103)		
In a relationship	n = 172	56.8%
Not in a relationship	n = 131	43.2%
Religion		
Jewish	n = 298	98.3%
Muslim	n = 5	1.7%
**Health-Related Factors**		
BMI (n = 299)	25.5	6.2
Physical activity frequency (n = 298)		
None (0 days/week)	n = 114	38.3%
Low (1–2 days/week)	n = 91	30.5%
Moderate (3–4 days/week)	n = 64	21.5%
High (5–7 days/week)	n = 29	9.7%
Sleep duration and quality * (n = 282)	2.72	1.33
**Mental Health Indicators**		
Depression: DHQ-9 * (n = 286)	11.5	6.8
Anxiety: GAD-7 * (n = 290)	10.1	5.5
**Dietary Pattern Indicators**		
Mediterranean diet * (n = 253)	6.2	2.2
Processed food consumption ** (n = 253)	7.7	5.1

* Score. ** Servings. SD = Standard Deviation.

**Table 2:** Relationships between the study variables.

	Mediterranean Diet	Processed Food	Sleep Disturbance	Age
Processed Food	−0.438 ***			
Physical Activity	0.272 ***		−0.136 *	
Anxiety	−0.202 **	0.141 *	0.148 *	−0.219 ***
Depression	−0.176 **	0.150 *	0.227 ***	−0.237 ***

* *p* < 0.05, ** *p* < 0.01, *** *p* < 0.001.

**Table 3:** Differences between low and high anxiety and depression scores.

Variable	PHQ-9 Depression Scale Low n = 124, High n = 162				*t* (df)	*p* Value
	Low		High			
	M	SD	M	SD		
Age	34.9	11.8	30.9	10.2	2.996 (238.884)	0.003
Physical activity (days/week)	2.0	2.0	1.6	1.8	1.663 (284)	0.097
BMI	25	5.1	25.9	6.7	−1.241 (279.783)	0.216
Mediterranean diet scale	6.5	2.3	5.9	2.1	2.250 (227.783)	0.025
Processed food consumption					−1.440 (248)	0.151
Sleep scale	2.4	1.2	2.9	1.4	−3.592 (268)	<0.001
	GAD-7 Anxiety Scale Low n = 100, High n = 190					
Age	35.5	12.4	31.1	9.9	3.075 (164.777)	0.002
Physical activity (days/week)	1.9	1.9	1.7	1.9	0.544 (288)	0.587
BMI	25.3	5.3	25.5	6.4	−0.226 (284)	0.821
Mediterranean diet scale	6.7	2.3	5.9	2.1	2.690 (251)	0.008
Processed food consumption	6.8	4.9	8.1	5.1	−1.828 (248)	0.069
Sleep scale	2.5	1.3	2.8	1.3	−1.755 (272)	0.080

SD = Standard Deviation. df = Degrees of freedom.

**Table 4:** Hierarchical regression analyses predicting anxiety symptoms.

	Combined Diet			Mediterranean Diet Only			Processed Food Only		
	R^2^ (ΔR^2^)	β	95% CI	R^2^ (ΔR^2^)	β	95% CI	R^2^ (ΔR^2^)	β	95% CI
Model 1 ^a^	0.163 ***			0.163 ***			0.163 ***		
Model 2 ^b^	0.170 (0.007)			0.169 (0.006)			0.170 (0.007)		
Model 3 ^c^	0.194 * (0.025 *)			0.190 * (0.021 *)			0.189 * (0.019 *)		
Mediterranean diet		−0.093	(−0.611, 0.132)		−0.153 *	(−0.696, −0.071)		-----	-----
Processed food		0.101	(−0.041, 0.254)					0.142 *	(0.020, 0.282)
Model 4 ^d^	0.209 * (0.014 *)			0.204 * (0.014 *)			0.204 * (0.016 *)		
Mediterranean diet		−0.084	(−0.585, 0.154)		−0.146 *	(−0.675, −0.053)		-----	-----
Processed food		0.105	(−0.036, 0.257)		----	----		0.142 *	(0.021, 0.281)
Sleep		0.126 *	(0.009, 1.028)		0.123 *	(0.002, 1.014)		0.130 *	(0.029, 1.045)

^a^ Model 1: Demographics (age, gender, relationship status, and socioeconomic status). ^b^ Model 2: Model 1 + health-related behaviors (BMI and physical activity). ^c^ Model 3: Model 2 + dietary patterns. ^d^ Model 4: Model 3 + sleep quality. * *p* < 0.05, *** *p* < 0.001. Note: standardized beta coefficients (β) and 95% confidence intervals (CI) for dietary patterns and sleep quality are from Models 3 and 4. Combined diet models include both the Mediterranean diet and processed food variables simultaneously.

**Table 5:** Hierarchical regression analyses predicting depression symptoms.

	Combined Diet			Mediterranean Diet Only			Processed Food Only		
	R^2^ (ΔR^2^)	β	95% CI	R^2^ (ΔR^2^)	β	95% CI	R^2^ (ΔR^2^)	β	95% CI
Model 1 ^a^	0.199 ***			0.199 ***			0.199 ***		
Model 2 ^b^	0.214 (0.015)			0.212 (0.013)			0.214 (0.015)		
Model 3 ^c^	0.235 * (0.022 *)			0.226 * (0.014 *)			0.234 * (0.020 *)		
Mediterranean diet		−0.051	(−0.600, 0.282)		−0.126 *	(−0.756, −0.011)		----	----
Processed food		0.123	(−0.016, 0.335)		----	----		0.146 *	(0.034, 0.344)
Model 4 ^d^	0.281 *** (0.045 ***)			0.271 *** (0.045 ***)			0.280 *** (0.046 ***)		
Mediterranean diet		−0.035	(−0.538, 0.321)		−0.112	(−0.705, 0.022)		----	----
Processed food		0.130	(−0.002, 0.339)		----	----		0.146 *	(0.038, 0.339)
Sleep		0.223 ***	(0.528, 1.712)		0.221 ***	(0.521, 1.703)		0.225 ***	(0.539, 1.719)

^a^ Model 1: Demographics (age, gender, relationship status, and socioeconomic status). ^b^ Model 2: Model 1 + health-related behaviors (BMI and physical activity). ^c^ Model 3: Model 2 + dietary patterns. ^d^ Model 4: Model 3 + sleep quality. * *p* < 0.05, *** *p* < 0.001. Note: standardized beta coefficients (β) and 95% confidence intervals (CI) for dietary patterns and sleep quality are from Models 3 and 4. Combined diet models include both the Mediterranean diet and processed food variables simultaneously.

## Data Availability

The original contributions presented in the study are included in the article, further inquiries can be directed to the corresponding author.

## Appendix A. STROBE Statement—Checklist of Items That Should Be Included in Reports of Cross-Sectional Studies

	**Item No**	**Recommendation**	**Page** **No**
**Title and abstract**	1	(a) Indicate the study’s design with a commonly used term in the title or the abstract.	1
		(b) Provide in the abstract an informative and balanced summary of what was performed and what was found.	1
**Introduction**			
Background/rationale	2	Explain the scientific background and rationale for the investigation being reported.	2–3
Objectives	3	State specific objectives, including any prespecified hypotheses.	3
**Methods**			
Study design	4	Present key elements of study design early in the paper.	3
Setting	5	Describe the setting, locations, and relevant dates, including periods of recruitment, exposure, follow-up, and data collection.	3
Participants	6	(a) Give the eligibility criteria and the sources and methods of selection of participants.	4
Variables	7	Clearly define all outcomes, exposures, predictors, potential confounders, and effect modifiers. Give diagnostic criteria, if applicable.	4–5
Data sources/measurement	8	For each variable of interest, give sources of data and details of methods of assessment (measurement). Describe comparability of assessment methods if there is more than one group.	4–5
Bias	9	Describe any efforts to address potential sources of bias.	5–6
Study size	10	Explain how the study size was arrived at.	4
Quantitative variables	11	Explain how quantitative variables were handled in the analyses. If applicable, describe which groupings were chosen and why.	4–5
Statistical methods	12	(a) Describe all statistical methods, including those used to control for confounding.	5–6
		(b) Describe any methods used to examine subgroups and interactions.	5–6
		(c) Explain how missing data were addressed.	5–6
		(d) If applicable, describe analytical methods taking into account the sampling strategy.	5–6
		(e) Describe any sensitivity analyses.	5–6
**Results**			
Participants	13	(a) Report numbers of individuals at each stage of study, e.g., numbers of the potentially eligible, examined for eligibility, confirmed eligible, included in the study, completed follow-up, and analyzed.	6–7
		(b) Give reasons for non-participation at each stage.	N/A
		(c) Consider use of a flow diagram.	N/A
Descriptive data	14	(a) Give characteristics of study participants (e.g., demographic, clinical, and social) and information on exposures and potential confounders.	6–7
		(b) Indicate number of participants with missing data for each variable of interest.	6–7
Outcome data	15	Report numbers of outcome events or summary measures.	8–9
Main results	16	(a) Give unadjusted estimates and, if applicable, confounder-adjusted estimates and their precision (e.g., 95% confidence interval). Make clear which confounders were adjusted for and why they were included.	9–11
		(b) Report category boundaries when continuous variables are categorized.	6–7
		(c) If relevant, consider translating estimates of relative risk into absolute risk for a meaningful time period.	N/A
Other analyses	17	Report other analyses performed, e.g., analyses of subgroups and interactions and sensitivity analyses.	6–7
**Discussion**			
Key results	18	Summarise key results with reference to study objectives.	11–13
Limitations	19	Discuss the limitations of the study, taking into account sources of potential bias or imprecision. Discuss both the direction and magnitude of any potential bias.	13
Interpretation	20	Give a cautious overall interpretation of results considering objectives, limitations, multiplicity of analyses, results from similar studies, and other relevant evidence.	11–13
Generalizability	21	Discuss the generalizability (external validity) of the study results.	13
**Other information**			
Funding	22	Give the source of funding and the role of the funders for the present study and, if applicable, for the original study on which the present article is based.	N/A

## References

[1] Rong J., Wang X., Cheng P., Li D., Zhao D. (2025). Global, regional and national burden of depressive disorders and attributable risk factors, from 1990 to 2021: Results from the 2021 Global Burden of Disease study. Br. J. Psychiatry.

[2] Wu Y., Li X., Ji X., Ren W., Zhu Y., Chen Z., Du X. (2025). Trends in the epidemiology of anxiety disorders from 1990 to 2021: A global, regional, and national analysis with a focus on the sociodemographic index. J. Affect Disord..

[3] Vos T., Lim S.S., Abbafati C., Abbas K.M., Abbasi M., Abbasifard M., Abbasi-Kangevari M., Abbastabar H., Abd-Allah F., Abdelalim A. (2020). Global burden of 369 diseases and injuries in 204 countries and territories, 1990–2019: A systematic analysis for the Global Burden of Disease Study 2019. Lancet.

[4] Santomauro D.F., Herrera A.M.M., Shadid J., Zheng P., Ashbaugh C., Pigott D.M., Abbafati C., Adolph C., Amlag J.O., Aravkin A.Y. (2021). Global prevalence and burden of depressive and anxiety disorders in 204 countries and territories in 2020 due to the COVID-19 pandemic. Lancet.

[5] Denton E.G., Gellman M.D. (2020). Depression: Symptoms. Encyclopedia of Behavioral Medicine.

[6] Penninx B.W.J.H., Eikelenboom M., Giltay E.J., van Hemert A.M., Riese H., Schoevers R.A., Beekman A.T.F. (2021). Cohort profile of the longitudinal Netherlands Study of Depression and Anxiety (NESDA) on etiology, course and consequences of depressive and anxiety disorders. J. Affect. Disord..

[7] Firth J., Marx W., Dash S., Carney R., Teasdale S.B., Solmi M., Stubbs B., Schuch F.B., Carvalho A.F., Jacka F. (2019). The effects of dietary improvement on symptoms of depression and anxiety: A meta-analysis of randomized controlled trials. Psychosom. Med..

[8] Grajek M., Krupa-Kotara K., Białek-Dratwa A., Sobczyk K., Grot M., Kowalski O., Staśkiewicz W. (2022). Nutrition and mental health: A review of current knowledge about the impact of diet on mental health. Front. Nutr..

[9] Sadeghi O., Keshteli A.H., Afshar H., Esmaillzadeh A., Adibi P. (2021). Adherence to Mediterranean dietary pattern is inversely associated with depression, anxiety and psychological distress. Nutr. Neurosci..

[10] Zhang H., Li M., Mo L., Luo J., Shen Q., Quan W. (2024). Association Between Western Dietary Patterns, Typical Food Groups, and Behavioral Health Disorders: An Updated Systematic Review and Meta-Analysis of Observational Studies. Nutrients.

[11] Li Y., Lv M.-R., Wei Y.-J., Sun L., Zhang J.-X., Zhang H.-G., Li B. (2017). Dietary Patterns and Depression Risk: A Meta-Analysis. Psychiatry Res..

[12] Cangelosi G., Grappasonni I., Nguyen C.T.T., Acito M., Pantanetti P., Benni A., Petrelli F. (2023). Mediterranean Diet (MedDiet) and Lifestyle Medicine (LM) for Support and Care of Patients with Type II Diabetes in the COVID-19 Era: A Cross-Observational Study. Acta Biomed..

[13] Angelidi A.M., Kokkinos A., Katechaki E., Ros E., Mantzoros C.S. (2021). Mediterranean Diet as a Nutritional Approach for COVID-19. Metabolism.

[14] Mosher L.T., Bizerra C., Davies K., Seabrook J.A., Keathley J. (2024). Evaluation of a 4-Week Interdisciplinary Primary Care Cardiovascular Health Programme: Impact on Knowledge, Mediterranean Diet Adherence and Biomarkers. BMJ Nutr. Prev. Health.

[15] Godos J., Currenti W., Angelino D., Mena P., Castellano S., Caraci F., Galvano F., Del Rio D., Ferri R., Grosso G. (2020). Diet and mental health: Review of the recent updates on molecular mechanisms. Antioxidants.

[16] Liu T., Zhong S., Liao X., Chen J., He T., Lai S., Jia Y. (2015). A Meta-Analysis of Oxidative Stress Markers in Depression. PLoS ONE.

[17] Shivappa N., Hébert J.R., Veronese N., Caruso M.G., Notarnicola M., Maggi S., Stubbs B., Firth J., Fornaro M., Solmi M. (2018). The relationship between the dietary inflammatory index (DII®) and incident depressive symptoms: A longitudinal cohort study. J. Affect. Disord..

[18] Lassale C., Batty G.D., Baghdadli A., Jacka F., Sánchez-Villegas A., Kivimäki M., Akbaraly T. (2019). Healthy dietary indices and risk of depressive outcomes: A systematic review and meta-analysis of observational studies. Mol. Psychiatry.

[19] Sureda A., Bibiloni M.D.M., Julibert A., Bouzas C., Argelich E., Llompart I., Tur J.A. (2018). Adherence to the mediterranean diet and inflammatory markers. Nutrients.

[20] Deane K.H.O., Jimoh O.F., Biswas P., O’Brien A., Hanson S., Abdelhamid A.S., Fox C., Hooper L. (2021). Omega-3 and polyunsaturated fat for prevention of depression and anxiety symptoms: Systematic review and meta-analysis of randomized trials. Br. J. Psychiatry.

[21] Li Z., Li B., Song X., Zhang D. (2017). Dietary Zinc and Iron Intake and Risk of Depression: A Meta-Analysis. Psychiatry Res..

[22] Muscaritoli M. (2021). The impact of nutrients on mental health and well-being: Insights from the literature. Front. Nutr..

[23] Young L.M., Pipingas A., White D.J., Gauci S., Scholey A. (2019). A systematic review and meta-analysis of B vitamin supplementation on depressive symptoms, anxiety, and stress: Effects on healthy and “at-risk” individuals. Nutrients.

[24] Merlo G., Bachtel G., Sugden S.G. (2024). Gut Microbiota, Nutrition, and Mental Health. Front. Nutr..

[25] Chamberlain S.R., Cavanagh J., De Boer P., Mondelli V., Jones D.N.C., Drevets W.C., Cowen P.J., Harrison N.A., Pointon L., Pariante C.M. (2019). Treatment-resistant depression and peripheral C-reactive protein. Br. J. Psychiatry.

[26] Jacka F.N., O’Neil A., Opie R., Itsiopoulos C., Cotton S., Mohebbi M., Castle D., Dash S., Mihalopoulos C., Chatterton M.L. (2017). A Randomised Controlled Trial of Dietary Improvement for Adults with Major Depression (the ‘SMILES’ Trial). BMC Med..

[27] Lane M.M., Gamage E., Travica N., Dissanayaka T., Ashtree D.N., Gauci S., Lotfaliany M., O’Neil A., Jacka F.N., Marx W. (2022). Ultra-Processed Food Consumption and Mental Health: A Systematic Review and Meta-Analysis of Observational Studies. Nutrients.

[28] Parletta N., Zarnowiecki D., Cho J., Wilson A., Bogomolova S., Villani A., Itsiopoulos C., Niyonsenga T., Blunden S., Meyer B. (2019). A Mediterranean-style dietary intervention supplemented with fish oil improves diet quality and mental health in people with depression: A randomized controlled trial (HELFIMED). Nutr. Neurosci..

[29] Knesset Israel (2024). The Welfare and Labor Committee Approved an Amendment to the National Health Insurance Law Regarding the Continuity of Rights. Knesset News.

[30] Von Elm E., Altman D.G., Egger M., Pocock S.J., Gøtzsche P.C., Vandenbroucke J.P. (2014). The Strengthening the Reporting of Observational Studies in Epidemiology (STROBE) Statement: Guidelines for Reporting Observational Studies. Int. J. Surg..

[31] Milton K., Bull F.C., Bauman A. (2011). Reliability and validity testing of a single-item physical activity measure. Br. J. Sports Med..

[32] Buysse D.J., Reynolds C.F., Monk T.H., Berman S.R., Kupfer D.J. (1989). The Pittsburgh Sleep Quality Index: A new instrument for psychiatric practice and research. Psychiatry Res..

[33] Shochat T., Tzischinsky O., Oksenberg A., Peled R. (2007). Validation of the Pittsburgh Sleep Quality Index Hebrew translation (PSQI-H) in a sleep clinic sample. Isr. Med. Assoc. J..

[34] Abu-Saad K., Endevelt R., Goldsmith R., Shimony T., Nitsan L., Shahar D.R., Keinan-Boker L., Ziv A., Kalter-Leibovici O. (2019). Adaptation and predictive utility of a Mediterranean diet screener score. Clin. Nutr..

[35] Spitzer R.L., Kroenke K., Williams J.B.W., Löwe B. (2006). A Brief Measure for Assessing Generalized Anxiety Disorder: The GAD-7. Arch. Intern. Med..

[36] Kroenke K., Spitzer R.L., Williams J.B.W. (2001). The PHQ-9: Validity of a Brief Depression Severity Measure. J. Gen. Intern. Med..

[37] Kroenke K., Spitzer R.L., Williams J.B.W., Monahan P.O., Löwe B. (2007). Anxiety Disorders in Primary Care: Prevalence, Impairment, Comorbidity, and Detection. Ann. Intern. Med..

[38] Manea L., Gilbody S., McMillan D. (2012). Optimal Cut-Off Score for Diagnosing Depression with the Patient Health Questionnaire (PHQ-9): A Meta-Analysis. CMAJ.

[39] Selvaraj R., Selvamani T.Y., Zahra A., Malla J., Dhanoa R.K., Venugopal S., Shoukrie S.I., Hamouda R.K., Hamid P. (2022). Association between dietary habits and depression: A systematic review. Cureus.

[40] Militao E.M.A., Uthman O.A., Salvador E.M., Vinberg S., Macassa G. (2024). Association between socioeconomic position of the household head, food insecurity and psychological health: An application of propensity score matching. BMC Public Health.

[41] Cui H., Xiong Y., Wang C., Ye J., Zhao W. (2024). The relationship between BMI and depression: A cross-sectional study. Front. Psychiatry.

[42] Pan A., Sun Q., Czernichow S., Kivimaki M., Okereke O.I., Lucas M., Manson J.E., Ascherio A., Hu F.B. (2012). Bidirectional association between depression and obesity in middle-aged and older women. Int. J. Obes..

[43] Chang de Pinho I., Giorelli G., Oliveira Toledo D. (2024). A narrative review examining the relationship between mental health, physical activity, and nutrition. Discov. Psychol..

[44] Mahindru A., Patil P., Agrawal V. (2023). Role of Physical Activity on Mental Health and Well-Being: A Review. Cureus.

[45] White R.L., Vella S., Biddle S., Sutcliffe J., Guagliano J.M., Uddin R., Burgin A., Apostolopoulos M., Nguyen T., Young C. (2024). Physical activity and mental health: A systematic review and best-evidence synthesis of mediation and moderation studies. Int. J. Behav. Nutr. Phys. Act..

[46] Ellis A., Jung S.E., Palmer F., Shahan M. (2022). Individual and interpersonal factors affecting dietary intake of community-dwelling older adults during the COVID-19 pandemic. Public Health Nutr..

[47] Gariépy G., Honkaniemi H., Quesnel-Vallée A. (2016). Social support and protection from depression: Systematic review of current findings in Western countries. Br. J. Psychiatry.

[48] Vaingankar J.A., Abdin E., Chong S.A., Shafie S., Sambasivam R., Zhang Y.J., Chang S., Chua B.Y., Shahwan S., Jeyagurunathan A. (2020). The association of mental disorders with perceived social support, and the role of marital status: Results from a national cross-sectional survey. Arch. Public Health.

[49] Hayes A.F., Rockwood N.J. (2017). Regression-based statistical mediation and moderation analysis in clinical research: Observations, recommendations, and implementation. Behav. Res. Ther..

[50] Radkhah N., Rasouli A., Majnouni A., Eskandari E., Parastouei K. (2023). The effect of Mediterranean diet instructions on depression, anxiety, stress, and anthropometric indices: A randomized, double-blind, controlled clinical trial. Prev. Med. Rep..

[51] Yin W., Löf M., Chen R., Hultman C.M., Fang F., Sandin S. (2021). Mediterranean diet and depression: A population-based cohort study. Int. J. Behav. Nutr. Phys. Act..

[52] Arteaga-Henríquez G., Simon M.S., Burger B., Weidinger E., Wijkhuijs A., Arolt V., Birkenhager T.K., Musil R., Müller N., Drexhage H.A. (2019). Low-grade inflammation as a predictor of antidepressant and anti-inflammatory therapy response in MDD patients: A systematic review of the literature in combination with an analysis of experimental data collected in the EU-MOODINFLAME consortium. Front. Psychiatry.

[53] Husain M.I., Foster J.A., Mason B.L., Chen S., Zhao H., Wang W., Rotzinger S., Rizvi S., Ho K., Lam R. (2023). Pro-inflammatory markers are associated with response to sequential pharmacotherapy in major depressive disorder: A CAN-BIND-1 report. CNS Spectr..

[54] Da Rocha B.R.S., Rico-Campà A., Romanos-Nanclares A., Ciriza E., Barbosa K.B.F., Martínez-González M.Á., Martín-Calvo N. (2021). Adherence to Mediterranean diet is inversely associated with the consumption of ultra-processed foods among Spanish children: The SENDO project. Public Health Nutr..

[55] Tristan Asensi M., Pagliai G., Lotti S., Corrao A., Colombini B., Giangrandi I., Sofi F., Dinu M. (2023). Adherence to the Mediterranean diet and ultra-processed foods consumption in a group of Italian patients with celiac disease. Nutrients.

[56] Loke G.M.I., Low J., Danaher J. (2023). The Influence of the Mediterranean Diet on Mood States, Anxiety, and Depression. Proc. Nutr. Soc..

[57] Winiarska-Mieczan A., Kwiecień M., Jachimowicz-Rogowska K., Donaldson J., Tomaszewska E., Baranowska-Wójcik E. (2023). Anti-Inflammatory, Antioxidant, and Neuroprotective Effects of Polyphenols—Polyphenols as an Element of Diet Therapy in Depressive Disorders. Int. J. Mol. Sci..

[58] Scott A.J., Webb T.L., Martyn-St James M., Rowse G., Weich S. (2021). Improving sleep quality leads to better mental health: A meta-analysis of randomised controlled trials. Sleep. Med. Rev..

[59] Zhai L., Zhang H., Zhang D. (2015). Sleep duration and depression among adults: A meta-analysis of prospective studies. Depress. Anxiety.

[60] Chavda S., Sarwar A., Batchelor-Parry H., Pankhania K., Upthegrove R. (2024). Adherence to a Mediterranean Diet and Impact on Mental Health Outcomes in Adolescents and Adults With Severe Mental Illness: A Systematic Review. BJPsych Open.

[61] Di Nucci A., Silano M., Cardamone E. (2025). Adherence to Mediterranean diet and health outcomes in adolescents: An umbrella review. Nutr. Rev..

[62] Bakırhan H., Pehlivan M., Özyürek F., Özkaya V., Yousefirad N. (2022). Diet, sleep and depression: Does adherence to the Mediterranean diet matter?. J. Turk. Sleep. Med..

[63] Samuthpongtorn C., Nguyen L.H., Okereke O.I., Wang D.D., Song M., Chan A.T., Mehta R.S. (2023). Consumption of ultraprocessed food and risk of depression. JAMA Netw. Open.

[64] Gustavson K., Knudsen A.K., Nesvåg R., Knudsen G.P., Vollset S.E., Reichborn-Kjennerud T. (2018). Prevalence and stability of mental disorders among young adults: Findings from a longitudinal study. BMC Psychiatry.

[65] Twenge J.M., Cooper A.B., Joiner T.E., Duffy M.E., Binau S.G. (2019). Age, period, and cohort trends in mood disorder indicators and suicide-related outcomes in a nationally representative dataset, 2005–2017. J. Abnorm. Psychol..

[66] Dopelt K., Houminer-Klepar N. (2025). The Impact of Social Media on Disordered Eating: Insights from Israel. Nutrients.

[67] Hjetland G.J., Finserås T.R., Sivertsen B., Colman I., Hella R.T., Andersen A.I.O., Skogen J.C. (2024). Digital self-presentation and adolescent mental health: Cross-sectional and longitudinal insights from the “LifeOnSoMe”-study. BMC Public Health.

[68] Houminer Klepar N., Davidovitch N., Dopelt K. (2024). Emotional eating among college students in Israel: A study during times of war. Foods.

[69] Maurer J., Meyrose A.-K., Kaman A., Mauz E., Ravens-Sieberer U., Reiss F. (2023). Socioeconomic Status, Protective Factors, and Mental Health Problems in Transition from Adolescence to Emerging Adulthood: Results of the Longitudinal BELLA Study. Child Psychiatry Hum. Dev..

[70] Pedroni G., Camerini A.-L. (2024). The importance of a healthy lifestyle to prevent mental health problems during crisis situations: Evidence from Corona Immunitas Ticino. J. Public Health.

[71] Keawtep P., Sungkarat S., Boripuntakul S., Sa-Nguanmoo P., Wichayanrat W., Chattipakorn S.C., Worakul P. (2024). Effects of Combined Dietary Intervention and Physical-Cognitive Exercise on Cognitive Function and Cardiometabolic Health of Postmenopausal Women with Obesity: A Randomized Controlled Trial. Int. J. Behav. Nutr. Phys. Act..

[72] Xiang M.Q., Liao J.W., Huang J.H., Deng H.L., Wang D., Xu Z., Hu M. (2019). Effect of a Combined Exercise and Dietary Intervention on Self-Control in Obese Adolescents. Front. Psychol..

